# Combining machine learning and iterative experiments to keep pace with emerging viral variants of concern

**DOI:** 10.1371/journal.pcbi.1014394

**Published:** 2026-06-17

**Authors:** Thomas Sheffield, Ryan C. Bruneau, Stephen Won, Kenneth L. Sale, Brooke Harmon, Le Thanh Mai Pham

**Affiliations:** 1 Biosecurity and Bioassurance, Sandia National Laboratories, Livermore, California, United States of America; 2 Biotechnology and Bioengineering, Sandia National Laboratories, Livermore, California, United States of America; 3 Bioresource and Environmental Security, Sandia National Laboratories, Livermore, California, United States of America; University of York, UNITED KINGDOM OF GREAT BRITAIN AND NORTHERN IRELAND

## Abstract

Modeling and predicting viral mutations before they emerge plays a crucial role in pandemic preparedness, enabling the early identification of emerging variants of concern (VOCs) and guiding timely updates to vaccines, diagnostic tests, and therapeutic strategies. However, existing machine learning models and large-scale experiments lose their predictive power as viral variants evolve further from the original strains in sequence space. Here, we present a scalable framework that integrates random forest and neural network machine learning models with targeted high-throughput experimentation to anticipate and evaluate emerging SARS-CoV-2 receptor-binding domain (RBD) variants. Using public datasets, we trained predictive models for binding to human Angiotensin-converting enzyme 2 (ACE2), RBD expression, and antibody escape, and refined these models through iterative integration of experimental data focused on over 200 variants derived from wild-type (WT) and Omicron strains. Through an indirect transfer learning approach, our machine learning models achieved high accuracy having correlation coefficients of up to 0.79 for antibody binding. The models were also generalizable across diverse antibody types including heavy-chain-only antibodies (HCAbs) by encoding complementarity-determining regions (CDRs) as input features. This dynamic approach enables rapid assessment of emerging variants, facilities prioritization of the therapeutic strategies, and supports a proactive, data-driven response to evolving viral threats.

## Author summary

The COVID-19 pandemic highlighted the threat posed by rapidly evolving viruses. SARS-CoV-2, the virus that causes COVID-19, continues to mutate in ways that can reduce the effectiveness of neutralizing antibodies from vaccines or prior infection. Predicting which viral variants might escape immune detection and identifying antibodies that can still work against them remains a major challenge. In this study, we developed a machine learning framework that helps forecast how mutations in the virus’s spike protein affect its ability to bind to human cells and avoid antibody recognition. We trained our models using large public datasets and then improved them with targeted lab experiments. Instead of treating each dataset separately, we used predictions from public data as features for training new models - a method known as indirect transfer learning. This allowed us to identify and validate antibodies with strong binding to emerging variants. Our approach supports faster, data-driven responses to viral evolution and can be applied to future outbreaks.

## Introductions

The COVID-19 pandemic, caused by the SARS-CoV-2 virus, profoundly affected global health and the economy, driving unprecedented efforts to mitigate its impact. One critical area of focus has been the discovery and development of neutralizing antibodies, which play a key role in preventing and treating infection. These antibodies target the SARS-CoV-2 spike protein, blocking its ability to bind the ACE2 receptor on human cells and replicate. However, the rapid evolution of SARS-CoV-2, with the emergence of variants carrying mutations in the spike protein, has posed significant challenges to ensuring the sustained efficacy of these antibodies [1].

Despite significant advances [2–4] in understanding the immune escape mechanisms of SARS-CoV-2, predicting the effectiveness of neutralizing antibodies against its mutated variants remains a complex and evolving challenge. Variants of concern, such as Delta, Omicron, and their sub-lineages, have mutations in the RBD of the spike protein, the primary target of neutralizing antibodies. These mutations alter the structure of the antibody binding domains of the spike protein and reduce the binding affinity of antibodies generated through prior infection or vaccination. The rapid emergence of such variants outpaced development of predictive models, which must account for the dynamic interplay between viral evolution and immune responses. This underscored the need for continuous surveillance, development of broad-spectrum vaccines, and advanced computational tools to anticipate potential escape mutations and assess their impact on antibody efficacy.

A few computational models have been developed to predict the binding of antibodies to mutations in SARS-CoV-2 [5]. These methods utilized structural modeling, machine learning, and evolutionary data to evaluate how changes in the viral spike protein, particularly in the RBD, affected antibody binding affinity. Molecular docking simulations, sequence-based prediction models, and deep learning frameworks have been employed to identify escape mutations that reduced neutralization efficacy [6–8]. Despite these advances, it is still challenging to predict the impact of mutations on viral pathogenicity and on binding affinity of neutralizing antibodies.

**Fig 1:**
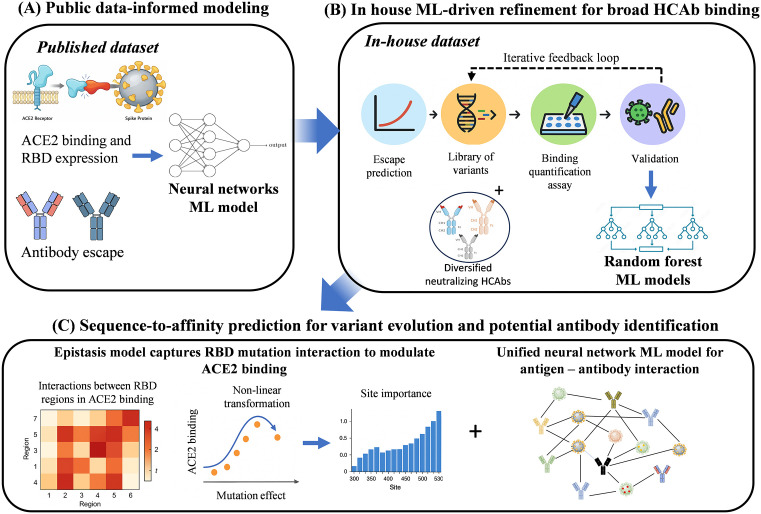
Indirect transfer learning framework for modeling SARS-CoV-2 variant evolution and antibody identification. The framework consists of three components: **(A)** Public data-informed models: These models predict ACE2 binding expression, and antibody escape of RBD variants of SARS-CoV-2, and the predictions are used to establish a library of potential variants for further analysis in step **B. (B)** ML-Driven Experimental Design and Model Validation: This component enables iterative refinement of models focused on ACE2-antibody interactions and antibody resistance, guiding targeted experimental validation. **(C)** Integration of Deep Mutational Data: This step incorporates deep mutational data and epistatic interactions to predict the impact of RBD sequence variants on ACE2 binding and antibody escape, resulting in a unified neural network model that predicts both the effects of single mutations and higher-order interactions. This figure is original and was created entirely by the authors; all graphical components and layout were prepared by the authors, and no third-party copyrighted images or clipart were used.

In this study, we present a combined experimental and machine learning framework designed to predict emerging SARS-CoV-2 variants of concern (VOCs) and identify potential neutralizing antibodies capable of countering VOCs as they arise (Fig 1). A key innovation of this approach is the use of indirect transfer learning, where predictions from machine learning models trained on large-scale public datasets such as ACE2 binding, RBD expression, and antibody escape are used as features to guide new models trained on in-house experimental data. This strategy enables the transfer of predictive knowledge between datasets with different endpoints or experimental conditions, overcoming common limitations in data compatibility. The framework uniquely integrates this indirect knowledge transfer with iterative, small-scale validation experiments, allowing for continuous model refinement. Unlike traditional models that treat receptor binding and antibody escape as separate tasks, our approach jointly models these interactions, providing a holistic assessment of viral pathogenicity and immune evasion [4,9]. The active-learning cycle further enhances adaptability, incorporating new experimental results into successive training rounds. Together, these features enable a more flexible and anticipatory response to emerging variants than is possible with conventional static or retrospective modeling pipelines [10,11].

## Results

We developed a suite of machine-learning models to integrate public deep mutational scanning data with in-house antibody binding measurements. These models were used iteratively to guide variant selection and experimental validation. Table 1 summarizes all machine-learning models used in this study, their data sources, predictive targets, architectures, and validation strategies. Public-data neural network models (“PEx_NN”, “PACE_NN”, “PAnti_NN”) were trained on published datasets (“PEx”, “PACE”, “PAnti”) to predict RBD expression, ACE2 binding, and antibody escape, respectively. In-house Random Forest models (“I1_RF”, “I2_RF”, “I3_RF”) were trained on successive experimental datasets (“I1”, “I2”, I3”) to predict antibody binding affinities. These models used predictions from PAnti_NN to augment their feature set. Com_NN is an alternative neural network model for antibody binding that combines PAnti and I3 directly instead of indirectly. Com_Epi is an epistasis model for ACE2 binding that combines PACE and experimental data to infer single mutation effects. All models were trained and evaluated using consistent data splits and validation procedures as described below. Detailed performance results are reported in the Results section.

**Table 1:** Overview of machine learning models developed in this study.

Model	Data Source	Predictive Target	Model Type / Framework	Objective Function	Key Input Features	Training & Validation Procedure
**PEx_NN**	Public dataset from Starr et al. (2020)	RBD expression (Δ mean fluorescence)	Fully connected NN (Keras/TensorFlow)	Mean-squared error (MSE)	One-hot encoded RBD mutations (res. 331–531)	Five-fold CV; 60 architectures randomly sampled; best by lowest RMSE; final model retrained on entire dataset
**PACE_NN**	Public dataset from Starr et al. (2020)	ACE2 binding (log₁₀(KD_variant/KD_WT))	Fully connected NN (Keras/TensorFlow)	MSE	One-hot encoded RBD mutations (res. 331–531)	Same as PEx_NN
**PAnti_NN**	Public dataset from Greaney et al. (2021)	Antibody escape (log₁₀ escape fraction)	Fully connected NN (Keras/TensorFlow)	MSE	One-hot encoded RBD mutations + one-hot encoded antibody identifiers (10 Abs)	Same as PEx_NN
**I1_RF**	In-house Exp 1 (HCAb × 7 variants)	HCAb binding log₁₀(KD_variant/KD_WT)	Random Forest (R Ranger v0.17.0)	Estimated Response Variance	16 CDR descriptors (8 SOCN + 8 Dragon) + predictions from PAnti_NN (Cov2-A2050, Cov2-A2082)	Five-fold CV; parameters fixed (500 trees, default depth)
**I2_RF**	In-house Exp 1 + 2 (15 HCAbs × 3 variants)	HCAb binding log₁₀(KD_variant/KD_WT)	Random Forest (R Ranger v0.17.0)	Estimated Response Variance	Same 16 CDR descriptors + 2 PAnti_NN features as I1_RF	Five-fold CV
**I3_RF**	In-house Exp 1–3 (5 HCAbs × 213 variants)	HCAb binding log₁₀(KD_variant/KD_WT)	Random Forest (R Ranger v0.17.0)	Estimated Response Variance	16 CDR descriptors + 10 PAnti_NN escape predictions	Five-fold CV
**Com_NN**	Combined I3 + PAnti datasets	HCAb binding + escape (merged log endpoints)	Fully connected NN (Keras, 2 layers [128, 32])	MSE	One-hot variant features + 16 CDR descriptors + dataset indicator	Five-fold CV
**Com_Epi**	Combined PACE + IACE datasets	ACE2 binding (log₁₀(KD_variant/KD_WT))	Global epistasis model (adapted from Starr et al.)	Least-squares fit on latent mutation effects	Latent per-mutation coefficients summed per variant	Five-fold CV

### Establishing a library of combinatorial mutations in SARS-CoV-2 sequences

We leveraged ACE2 binding to SARS-CoV-2 RBD and RBD expression data from a previously published paper [11] to develop the PACE_NN and PEx_NN machine learning models**.** The expression data included 135,386 unique sequences and the binding data included 105,526 unique sequences. For both datasets over 90% of unique sequences were five or fewer mutations away from the Wildtype (WT) strain and the majority (59% for expression and 62% for binding) of unique sequences were 2 or 3 mutations away from WT. Endpoints for the binding model were the Log_10_ (K_D___variant_/K_D_WT_), also called the delta (Log_10_(K_A_), of each variant, where K_D_ is the dissociation constant of binding to the ACE2 receptor. Expression endpoints were the mean fluorescence of each variant relative to the WT strain. Published antibody binding escape data [12] were used to develop the PAnti_NN model for antibody escape, where the modeling endpoint was the Log_10_(Binding escape Fraction) for each variant and antibody combination (S1 Fig). The final tuned PACE_NN, PEx_NN, and PAntiNN models showed a cross-validated Q^2^ of 0.92, 0.86, and 0.66, respectively. Here, $Q^{\text{2}}=\;\frac{\sum {\left(y_{i}-f_{i}\right)}^{\text{2}}}{\sum \left(y_{i}-\overset{―}{y}\right)}$, where $y_{i}$ are actual endpoints and $f_{i}$ ‘s are predicted endpoints. PEx_NN and PACE_NN were additionally tuned inside the cross-validation loop to achieve a more accurate estimation of the tuned models’ accuracies; this means that the tuning process occurred separately inside each cross-validation fold so that overfitting due to tuning would be accounted for. When tuned inside the loop, the cross-validated Q^2^ for PACE_NN and PEx_NN were slightly less than before, equaling 0.91 and 0.84, respectively. PAnti_NN was not additionally tuned in the loop due to computational constraints. Final models were trained on the entire dataset using their respective tuned architectures and used to make predictions of ACE2 binding, expression and antibody binding for all variants with two or fewer mutations in the RBD relative to the WT strain, and VOCs such as Omicron BA.1 and Omicron BA.5 strains.

Model predictions from PEx_NN, PACE_NN, and Panti_NN were integrated to guide the selection of variants for the third experimental round. Variants predicted to have greater or equal ACE2 binding and expression than their parent strain while exhibiting increased antibody-escape potential were prioritized, yielding a focused panel of 213 RBD variants spanning Omicron BA.1, BA.5, and related single- and combinatorial mutations. These model-guided variants were then produced in HEK cells, and their binding affinities with ACE2 and five representative HCAbs were experimentally characterized [12,13].

### Training and Validating Antibody-Binding Models Using Circulating and Potential SARS-CoV-2 RBD Variants

**Fig 2:**
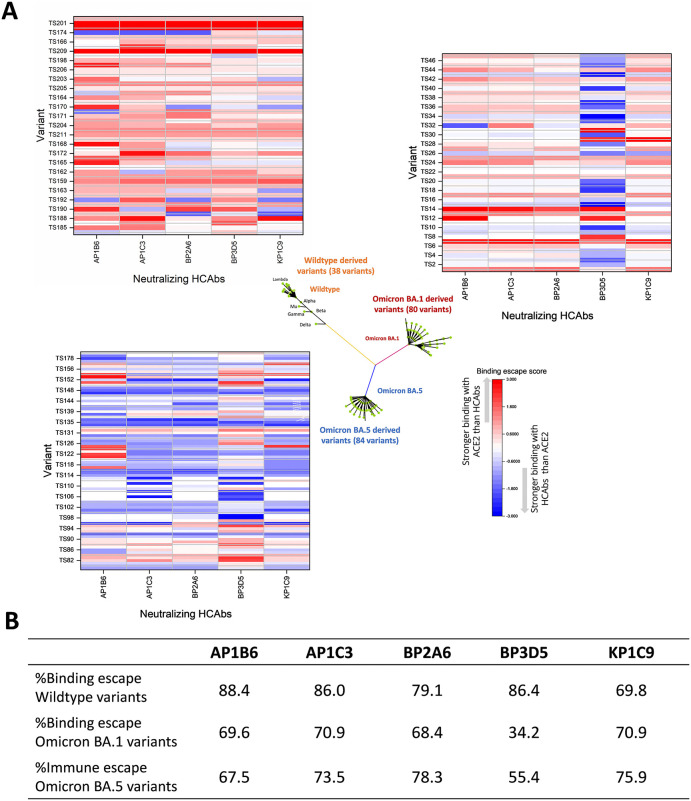
Comparative binding analysis of potential SARS-CoV-2 variants to the human ACE2 receptor and selected heavy-chain-only antibodies (HCAbs). **(A)** Binding affinity profiles of SARS-CoV-2 variants interacting with the human ACE2 receptor and five representative HCAbs measured via high-throughput binding assays. Data includes 38 variants derived from wildtype (upper left), 80 variants derived from Omicron BA.1 (upper right) and 84 variants derived from Omicron BA.5 (lower left). Data points represent averaged measurements from triplicate experiments. **(B)** Percentage of variants with a binding escape score, log10(KD_HCAbs/KD_ACE2) > 0, indicating that variant RBDs are predicted to bind human ACE2 with higher affinity than the tested HCAbs. This threshold was used for screening predicted escape potential and not as a definitive threshold for biologically significant immune escape.

We generated RBD variants derived from WT, Omicron BA.1, and Omicron BA.5 strains of SARS-CoV-2 and assessed their binding to human ACE2 and a panel of five neutralizing HCAbs (Fig 2). Binding escape scores and inhibition profiles were used to characterize variant-specific antibody resistance. A binding escape score > 0 was used as a permissive screening cutoff to identify variants with predicted escape potential, not as a strict threshold for biologically meaningful immune escape. The binding escape scores Log_10_ (K_D___HCAbs_/K_D_ACE2_) for 202 variants derived from WT, Omicron BA.1, and Omicron BA.5 were presented as a heat map in Fig 2A where the color scale goes from blue to red with red being the highest escape score. The data showed that even with the best antibody, BP3D5, 86.4% of variants derived from WT escaped HCAb binding, 34.2% from Omicron BA.1 escaped HCAb binding, and 55.4% escaped HCAb binding when they were derived from Omicron BA.5 (Fig 2B).

### Refinement of machine learning models using iterative experimental datasets

**Fig 3:**
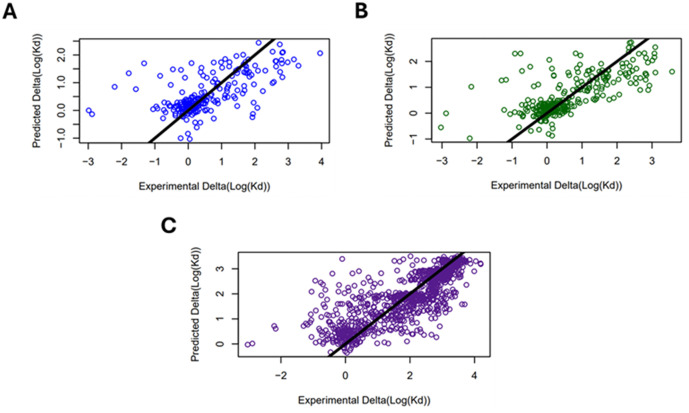
Comparison between experimental and predicted binding affinities expressed as Log10(KD_variant/ KD_WT) across three successive Random Forest (RF) models trained with progressively larger datasets. **(A)** I1_RF: trained on binding data for 59 newly characterized HCAbs against the Beta, Delta, Gamma, Lambda, Mu, Omicron BA.1, and WT variants (RMSE = 0.81; Corr = 0.63; Q² = 0.40). **(B)** I2_RF: extended to 15 antibodies tested against Omicron BA.1, Omicron BA.5, and WT (RMSE = 0.78; Corr = 0.69; Q² = 0.48). **(C)** I3_RF: final model trained on five representative HCAbs evaluated across 213 prioritized RBD variants (RMSE = 0.72; Corr = 0.82; Q² = 0.67). Model performance improved consistently as additional ‌‌experimental data were incorporated.

New machine learning models of antibody binding were refined using experimental datasets collected in this study. The refined random forest (RF) models are referred to as I1_RF, I2_RF, and I3_RF. I1_RF consisted of binding data for 59 HCAbs to the Beta, Delta, Gamma, Lambda, Mu, Omicron BA.1, and WT variants. I2_RF included binding data for the top 15 HCAbs to Omicron BA.1, Omicron BA.5, and WT [13]. I3_RF included binding data for 5 HCAbs to the 213 variants selected using PEx_NN, PACE_NN, and PAnti_NN (S1 Table). The Q^2^ for I1_RF, I2_RF, and I3_RF were 0.40, 0.48, and 0.67, respectively (Fig 3). As an alternative comparison, one fifth of the variants only found in the I3 dataset were held out as a test set for models trained on the I1, I2, and remainder of the I3 dataset. The Pearson correlations for I1_RF, I2_RF, and I3_RF tested on this set were 0.37, 0.46, and 0.64, respectively (S2 Fig). To take advantage of the larger public datasets while avoiding the difficulties introduced due to the different endpoint measurements, the focus on mutations of the WT strain, and differences in experimental methods, the predictions of PAnti_NN were used as features for training the new models. Specifically, the predicted escape scores for a given variant and a selection of antibodies were used as features for the corresponding variant in this model, with one feature per selected antibody.

To verify that the refined antibody-binding model accurately predicts HCAb performance across emerging variants, we validated the final random forest model (I3_RF), which was trained on five HCAbs across 213 RBD variants, using both cross-validation and prospective experimental testing. Model predictions were used to prioritize candidate HCAbs expected to retain or improve binding breadth. Based on I3_RF predictions across the prioritized RBD variant panel, HCAbs DP4F2, BP2A3, BP2G12, and AP1B1 were selected as candidate antibodies predicted to retain broader binding across the tested variants. These four HCAbs were then carried forward for experimental evaluation, where their binding performance was assessed against the prioritized variant set.

### Identifying broadly neutralizing HCAbs Using model-guided selection

**Fig 4:**
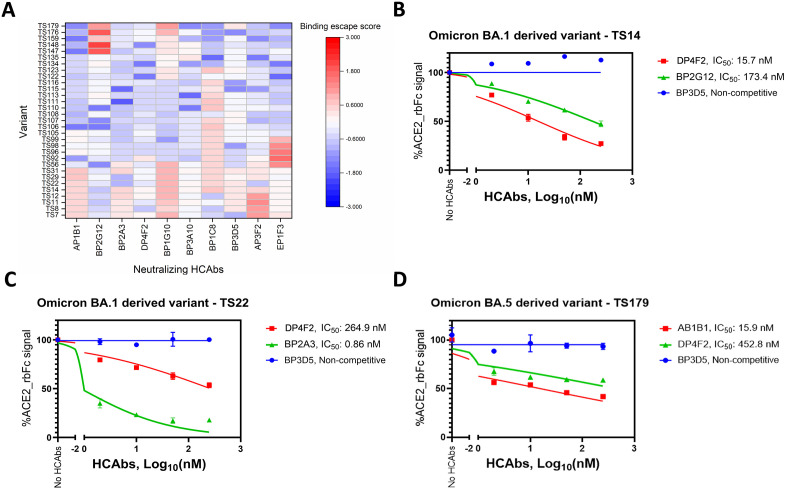
Binding escape analysis of Omicron-derived variants against HCAbs. **(A)** Heatmap of binding escape scores for Omicron-derived spike variants against a panel of 10 representative neutralizing HCAbs. **(B-D)** Inhibition curves showing % hACE2-rbFc signal vs. Log10 HCAb concentration for three representative variants. Binding escape score = Log_10_[K_D_HCAb_/K_D_ACE2_]. The percentage hACE2-rbFc signal was calculated by dividing the maximum response in binding with SARS-CoV-2 RBD of the premixed hACE2-rbFc and HCAbs by the maximum response of hACE2-rbFc in solo binding of the SARS-CoV-2 RBD variant, multiplied by 100. The IC_50_ values were processed and fitted into a non-linear regression curve using GraphPad Prism 9.0. The experiments were performed in triplicate, and the error bars are the standard deviation of the mean.

The I3_RF model successfully identified HCAbs from our broader library that exhibited stronger binding to the prioritized RBD variants than the five antibodies included in the original training set. Specifically, DP4F2, BP2A3, BP2G12, and AP1B1 were predicted and subsequently confirmed experimentally to display substantially enhanced binding across model-prioritized RBD variants (Fig 4A). Binding-escape score distributions for each HCAb are summarized as boxplots in S3 Fig. These boxplots highlight substantial heterogeneity in variant-level performance across the antibody panel.

To further elucidate the mechanism of action, we performed competition assays between the HCAbs and human ACE2 fused to a rabbit Fc domain (hACE2-rbFc) for binding to variant RBDs. HCAbs that compete with ACE2 reduced ACE2–RBD binding, indicating direct receptor-blocking potential. Fig 4B-4D shows representative inhibition curves for selected HCAb-variant combinations that illustrate distinct competition behaviors. Not all four selected HCAbs are shown in each panel, as these subplots were intended to present representative examples of competitive and non-competitive binding rather than every antibody-variant combination for each tested variant. Notably, DP4F2 and BP2G12 strongly competed with ACE2 for the Omicron BA.1-derived variant TS14 (Fig 4B), whereas DP2F2 and BP2A3 showed strong competition for the Omicron BA.1-derived variant TS22 (Fig 4C). Similarly, AP1B1 and DP4F2 blocked ACE2 binding to the Omicron BA.5-derived variant TS179 (Fig 4D). In contrast, BP3D5 did not exhibit competitive binding in any of these assays (Fig 4B-4D).

### Integrating multi-dataset binding measurements through a combined neural network model

**Fig 5:**
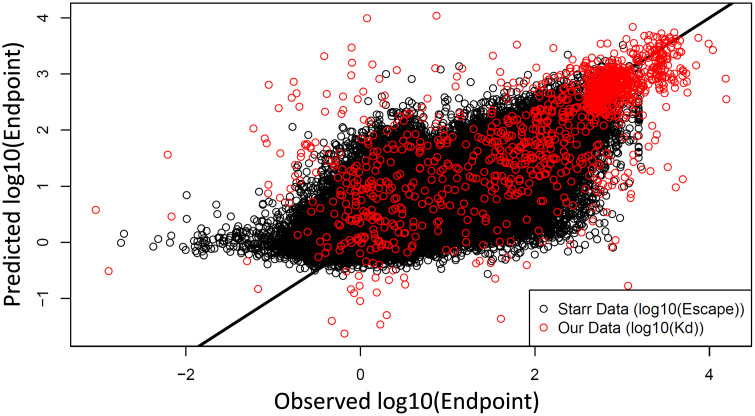
Performance of the Com_NN model. Scatter plot comparing observed and predicted SARS-CoV-2 antibody binding endpoints for the full PAnti and I3 datasets. Each point represents a variant and antibody pair, and the predictions are from the entire dataset after cross-validation. Black points are from the PAnti dataset, plotted as Log_10_[Binding escape_variant_/Binding escape_WT_]. Red points represent experimental measurements from our I3 dataset, shown as Log_10_[K_D_variant_/K_D_WT_], where K_D_ reflects the dissociation constant for antibody binding. The diagonal line indicates perfect concordance between the two datasets. Performance metrics for the combined dataset, where both data sources are split evenly among five cross-validation folds are: RMSE = 0.56, Corr = 0.79, Q^2^ = 0.62.

To complement the random forest–based binding model, we developed a fully connected neural network (Com_NN) designed to integrate antibody-binding data from two sources: (1) our I3 dataset, measured as Log₁₀(KD_variant/KD_WT), and (2) the published PAnti dataset, measured as Log₁₀(Binding escape_variant/Binding escape_WT) [11] (Fig 5). Because these datasets differ in both measurement scale and biological interpretation, we included a dataset-identity indicator as an explicit model feature. This strategy allowed the Com_NN model to account for systematic variation between datasets while leveraging shared signal related to antibody-RBD binding determinants. The integrated Com_NN model achieved an overall predictive performance of Q² = 0.62 across both datasets, compared to Q² = 0.66 when trained on the external dataset alone. When evaluated specifically on our I3 dataset, cross-validated performance metrics were RMSE = 0.92, Pearson correlation = 0.70, and Q² = 0.46 (red points in Fig 5). These results indicate that, although merging datasets introduces modest noise due to differing endpoint definitions, it enables improved generalization across antibody-variant combinations and supports prediction in binding regimes underrepresented in any single dataset.

We additionally built a global epistasis model (Com_Epi) to predict ACE2 binding using ACE2 binding measurements from both our I3 dataset and the PACE dataset [11]. Following the framework described by Starr et al., Com_Epi estimates ACE2 affinity as a non-linear transformation of a linear combination of single mutation effects. This model achieved strong overall performance across the combined dataset (RMSE = 0.55, Corr = 0.96, Q² = 0.92), but performance decreased when evaluated solely on our I3 data (RMSE = 0.98, Corr = 0.65, Q² = 0.36), reflecting the broader sequence and binding diversity present in our measurements.

**Fig 6:**
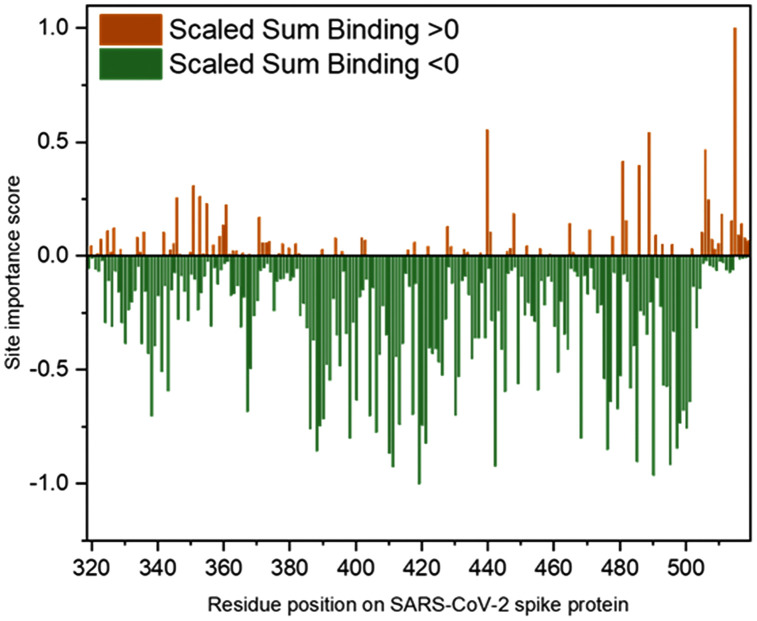
Positional importance of mutations affecting ACE2 binding based on Com_Epi. Bar plot summarizing the per-site impact of mutations across SARS-CoV-2 variants. For each position, positive (orange) and negative (green) single-mutation effects were extracted from the final model and summed separately, then scaled by the maximum absolute sum across all positions. This yields a normalized site-wise importance score. Additionally, residuals were calculated as the difference between predicted and observed Delta[Log₁₀(Kₐ)] values for ACE2 binding, capturing the contribution of epistatic interactions at each site.

**Fig 7:**
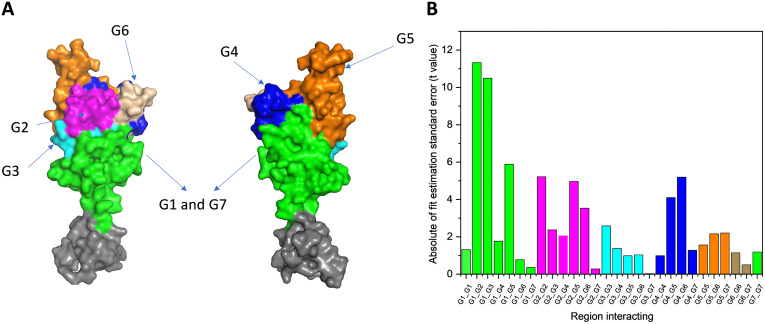
Interactions between RBD regions with an effect on ACE2 binding beyond single mutation effects. **(A)** Structural representation of the SARS-CoV-2 RBD protein colored by epitope clusters. Views are from different angles to highlight spatial distribution of antibody-binding regions. **(B)** The absolute value of the number of standard errors away from zero (the t value) represents the likelihood of a pair being genuinely predictive between regions on SARS-CoV-2 RBD protein.

From Com_Epi, we extracted per-mutation effects to identify RBD sites where substitutions consistently increase or decrease ACE2 binding (Fig 6) and evaluating performance through cross-validation (S4 Fig). These site-level effects provide a biologically interpretable view of how individual substitutions influence receptor engagement, and several highlighted positions fall within RBD regions previously implicated in ACE2 binding and antigenic change. Thus, Com_Epi is useful not only as a predictive model, but also as a framework for identifying mutation-sensitive positions associated with functional variation in receptor binding. To examine higher-order sequence interactions, we fit a linear model to the residuals of Com_Epi after accounting for single-mutation contributions. The RBD was partitioned into seven structural regions, and mutation counts within all 28 region-pair combinations were used to quantify epistatic interactions affecting ACE2 affinity (Fig 7). For each variant, we counted the number of mutation pairs that fell within each region-pair combination. These counts served as independent variables to explain the residual binding signal left unaccounted for by Com_Epi (Fig 7). These region-level interaction terms suggest that ACE2 binding is shaped not only by individual substitutions, but also by cooperative effects among RBD regions. Such non-additive interactions may help explain why models based only on single-mutation effects become less accurate for more sequence-divergent variants, including Omicron BA.1 and BA.5. The linear model fit to these individual variables was used to calculate the absolute value of the coefficient/standard error (t-value) for each pair of regions. The resulting coefficients highlight specific region-level interactions that modulate binding beyond what is explained by individual mutation effects.

## Discussion

We successfully developed the PEx_NN, PACE_NN, and PAnti_NN ML models for RBD expression, ACE2 binding, and antibody escape using publicly available datasets and employed them to guide targeted experiments, and data from these experiments was in turn used to refine the models. This iterative framework enabled adaptation to emerging SARS-CoV-2 variants, including those outside the sequence space of previously characterized datasets. The cycle can be repeated indefinitely to maintain alignment with ongoing viral evolution [11]. We implemented the global epistasis model in Starr et al. [11] on a combined PACE and I3 dataset and used fivefold cross-validation to estimate its accuracy. While its overall accuracy was on par with our PACE_NN model with respect to the public PACE dataset, it’s performance on our I3 dataset, containing variants with many more mutations, was poor. Our findings suggest that in sequence neighborhoods dominated by variants closely related to the WT strain, additive effects of single mutations are generally sufficient to explain the observed binding and expression patterns. However, the limitations of such single-mutation models became evident as more divergent variants such as Omicron BA.1 and BA.5 were incorporated into the dataset, necessitating more flexible and nonlinear modeling approaches. Com_Epi was useful not only for ACE2-binding prediction, but also for identifying mutation-sensitive RBD positions and higher-order regional interactions that may influence receptor engagement, particularly in more divergent variant backgrounds such as Omicron BA.1 and BA.5. A key advantage of our I3_RF modeling approach is its ability to generalize across antibody types. Whereas a new global epistasis model would have to be trained separately for each antibody, our framework supports simultaneous prediction across nanobodies, HCAbs, and full-length antibodies by encoding complementarity-determining region (CDR) sequences as input features. This allows us to assess and compare escape potential for multiple antibodies in parallel, eliminating the need for redundant model construction.

To inform experimental design in a way that supports pandemic preparedness, we generated variant selection criteria based on model predictions. Specifically, we focused on RBD variants derived from Omicron BA.1 and BA.5 that were predicted to exhibit enhanced antibody escape while maintaining minimum thresholds for ACE2 binding and RBD expression. This ensured that selected variants were both viable and of immunological concern. Experimental testing of these model-prioritized variants enabled (1) confirming whether the predicted variants could evade existing HCAbs and (2) assessing the ability of additional candidate antibodies to bind and neutralize variants of concern.

The I3_RF model also successfully identified new HCAbs with improved binding to model-prioritized variants. Four HCAbs (AP1B1, BP2G12, BP2A3, and DP4F2) were predicted to retain broader binding across the prioritized RBD variants and were experimentally confirmed to bind more effectively than members of the original panel. This combined computational and experimental strategy helped identify antibodies with improved cross-variant binding potential. Binding-escape score distributions (S1 Fig) highlight substantial heterogeneity across antibodies, with model-selected HCAbs avoiding the large escape outliers observed for several initial HCAbs. Together with EC₅₀ measurements, these results show that the newly identified HCAbs provide more consistent performance across diverse RBD variant backgrounds**,** supporting the utility of model-guided antibody selection for expanding coverage of the mutational landscape.

To improve model performance in sparse or underrepresented regions of sequence space, we developed a strategy of indirect transfer learning. The PAnti dataset [10,11] provided broad RBD variant coverage but limited antibody diversity and used relative escape scores instead of dissociation constants. Rather than merging these datasets directly, which would have introduced scale incompatibilities and endpoint inconsistencies, we trained models on the public data separately (Panti_NN) and used their predictions as input features for our own antibody escape models (I1-I3_RF). This hybrid strategy allowed us to benefit from the breadth of the public dataset while preserving the precision and interpretability of our targeted experimental measurements. When comparing indirect transfer learning to a baseline model trained directly on both datasets (Com_NN), we found that the indirect transfer learning model provided superior predictive accuracy on our I3 dataset. The final model, which leveraged features derived from prior predictions rather than raw data fusion, outperformed direct integration, underscoring the advantage of prediction-based transfer in cross-study learning scenarios.

This approach has broader implications for modeling less studied or newly emerging viruses within the same family or genus. By incorporating model predictions from well-characterized pathogens such as SARS-CoV-2 into models of related viruses (e.g., SARS-like betacoronaviruses), we can rapidly bootstrap predictive systems in low-data environments. Such models can support early-stage assessments of receptor binding, immune escape potential, and therapeutic effectiveness critical components of rapid response systems during zoonotic spillovers and emerging viral outbreaks.

## Conclusion

Our study demonstrates the power of combining high-throughput experimentation with machine learning to proactively address the challenges of viral evolution. By developing predictive models for ACE2 binding, RBD expression, and antibody escape and continuously refining them with new experimental data we established a scalable and adaptable framework capable of keeping pace with emerging SARS-CoV-2 variants. This framework is scalable in that it can incorporate newly generated data for additional variants, enabling the system to continuously learn from naturally evolved mutations. Our approach not only enables rapid identification of cross-variant neutralizing HCAbs but also allows for informed experimental design and risk assessment of potential immune escape mutations. Furthermore, through indirect transfer learning, we successfully adapted public datasets without compromising model performance, highlighting a practical strategy for integrating diverse data sources. In conclusion, this targeted, model-informed approach offers a scalable and adaptable framework for anticipating viral evolution and accelerating therapeutic development in the face of future outbreaks.

## Methods

### Data source and processing

There were six different datasets that were used to build machine learning models: three came from public datasets (“PEx”, “PACE”, “PAnti”) and four came from in-house experiments (“I1”, “I2”, “I3”, “IACE”). PEx and PACE were drawn from Starr et al. (11) and contained RBD expression and ACE2 binding data, respectively. PAnti was drawn from Greaney et al. (10) and contained antibody binding data for various antibodies. I1 came from our original antibody binding experiment. I1 was combined with a second experiment to make the I2 dataset, and the I2 dataset was combined with a third experiment to make the I3 dataset. Whereas I1, I2, and I3 consist of antibody binding data, IACE contains all of the ACE2 binding experiments that were performed in-house.

PEx, PACE, and PAnti were used as training data to build neural network models called “PEx_NN”, “PACE_NN”, and “PAnti_NN”, respectively. These three models were used together to select variants to be tested in our third experiment. I1, I2, and I3 were used as training data to build random forest models called “I1_RF”, “I2_RF”, and “I3_RF”, respectively. I1_RF, I2_RF, and I3_RF are hybrid models that incorporate the predictions of PAnti_NN into their feature sets. A neural network model called “Com_NN” was built as an alternative to I3_RF that uses the combined I3 and PAnti datasets to train a neural network directly. Lastly, an epistasis model called “Com_Epi” was trained on a combination of the PACE and IACE datasets to compare with the PACE_NN model and examine single mutation effects.

### Model architecture and training

#### Public data models: PEx_NN, PACE_NN, and PAnti_NN.

In the PEx, PACE, and PAnti datasets, the mean of endpoint was used when there were multiple measurements of the same variant. Only data points that passed the authors’ pre-count filter were used in the PAnti dataset. Only mutations on the RBD identified as locations 331–531 of the SARS-CoV2 spike protein were considered. RBD variant sequences were one-hot encoded, with one column for each mutation present in the dataset. For PAnti_NN, the antibodies being tested were also one-hot encoded, with nine columns representing ten antibodies.

We used a Keras/TensorFlow based fully connected neural network to fit PEx_NN, PACE_NN, and PAnti_NN. Each of these models was trained using the same process. The network architectures for each were chosen using a tuning process that randomly sampled sixty different architectures from a predetermined list of possible configurations and chose the architecture with the lowest fivefold cross-validated root-mean-square error (RMSE). Cross-validation was performed using five folds with variant-antibody pairs randomly distributed between them. The number of layers was chosen to be either 2 or 3 and the number of nodes in each layer was chosen from a power of two between 4 and 256. Potentially leaky ReLU activation layers were set after each layer with an alpha of either 0 or 0.1. Once an architecture was chosen, a final version of each model was trained using that architecture on the entire dataset.

In order to achieve a more accurate estimation of the final tuned models’ error, PEx_NN and PACE_NN were additionally “tuned-in-loop”, meaning that a separate tuning process was undergone for each cross-validation fold. For each fold, one fifth of the data was set aside as an independent test set, and the architecture tuning was performed on the remaining 80% of the data. This required sampling 60 different architectures for each fold, performing an inner fivefold cross-validation on that fold’s training data for each architecture, choosing the best architecture for that fold, training a model on the entire fold’s training data using that architecture, and returning the predictions of that final model for that fold. Thus, candidate architectures were evaluated only within the training portion of each outer fold, and the held-out outer fold was not used for model selection. Tuned-in-loop error statistics were computed by comparing the final predictions for each fold with the true values. This procedure did not affect the final models but was used only to obtain a less biased estimate of error by accounting for overfitting during tuning. Because tuned-in-loop evaluation was substantially more computationally expensive than regular tuning, the PAnti dataset was much larger than the PEx and PACE datasets and tuned-in-loop error statistics differed only slightly from tuned-out-of-loop estimates, PAnti_NN was not evaluated using tuned-in-loop cross-validation. Model quality was assessed using RMSE, Pearson correlation, and Q² = 1 − Variance/MSE.

PEx_NN and PACE_NN were trained and evaluated using fivefold cross-validation with variant-level splits, ensuring that no identical variants appeared in more than one-fold. PAnti_NN was split on the variant-antibody pair level, ensuring no variant-antibody pairs appeared in more than one-fold. Approximately 80% of samples were used for training and 20% for testing in each fold, with 20% of the training set reserved for validation during tuning. The dataset sizes were as follows: PEx_NN - 4038 variants entries; PACE_NN - 4012 variant entries and PAnti_NN - 34270 variants-antibody pairs.

#### Antibody binding models built with in-house Random-Forest models: I1_RF, I2_RF, and I3_RF.

I1_RF, I2_RF, and I3_RF were fit using random forest models on successive experimental datasets collected from this study, namely I1, I2, and I3. I1 included binding data for ACE2 and 59 HCAbs across seven SARS-CoV-2 variants: Beta, Delta, Gamma, Lambda, Mu, Omicron BA.1, and the ancestral WT strain [13]. I2 focused on a subset of the top 15 neutralizing HCAbs and included binding data for ACE2 and these antibodies against Omicron BA.1, Omicron BA.5, and WT. I3 comprised binding data for ACE2 and 5 HCAbs tested against 213 RBD variants previously selected through model-guided prioritization. Random forest models for successive versions were built with antibody binding Log_10_(K_D_variant_/K_D_WT_) as the endpoint [14]. HCAb-variant interaction pairs with poor K_D_ fitting or missing a corresponding K_D_WT_ were removed from the dataset and duplicated pairs were averaged together. The total number of unique datapoints available to train I1_RF, I2_RF, and I3_RF were 236, 280, and 1,183, respectively (S1 Table).

The HCAbs’ CDRs were combined into a single sequence and used to generate features with the R package protr [15] v1.7-5. Eight features were based on sequence-order-coupling numbers (SOCN) with a maximum lag of four, and eight features were scales-based descriptors based on a selection of ten Dragon [16] topological descriptors (namely, the Balaban- and Wiener-type index from Z, mass, van der Waals, electronegativity and polarizability weighted distance matrices). The top two principal components and a maximum lag of 2 were used. The Log_10_[Binding escape Fraction] predictions made by PAnti_NN for selected antibodies with the given variant were also used as features. Cov2-A2050 and Cov2-A2082 predictions were used as features in I1_RF and I2_RF, and all ten antibodies’ predictions were used as features in I3_RF. These predictions were added as features because the I1 and I2 datasets otherwise contained too few variants to featurize or account for them effectively. Since I3 contained many more variants, more antibody predictions could be used. Random forest models were built using the R Ranger package v0.17.0 with 500 trees and default parameters. Fivefold cross-validation was performed with antibody-variant pairs split randomly among the folds.

#### Antibody binding model built using combined datasets: Com_NN.

The PAnti dataset was combined with I3 and used to build a neural network model called Com_NN. The Log_10_[K_D_variant_/K_D_WT_] endpoint was used for I3 and the Log_10_[Binding escape Fraction] endpoint was used for the PAnti dataset, total approximately 35000 variant-antibody pairs. Antibody features were encoded in the same way as in I3_RF, using eight SOCN descriptors and 8 scales-based descriptors. Variant features were one-hot encoded by mutation. An additional one-hot feature identified which dataset a data point was from. The two datasets were each split evenly among five cross-validation folds. Machine learning was performed using a Keras fully connected neural network with 2 layers of size 128 and 32, each followed by an ReLU activation layer with α=0.

#### Epistasis model Com_Epi and region interactions.

Epistasis modeling code provided by Starr et al. [11] was adapted to fit the combined dataset consisting of PACE and IACE, yielding approximately 4000 variant entries. According to the authors this global epistasis model “fit regression models that represent the phenotype of each library variant as a sum of latent-scale effects of all component amino acid mutations, which are transformed by a flexible nonlinear curve to the observed experimental scale; the shape of the nonlinear curve and the single-mutant effect terms are fit simultaneously to all of the data” (11). We added a fivefold cross-validation loop to this code which randomly partitioned the mutations into different folds; this was used to estimate model accuracy. Single mutation effects were extracted from a final model trained on the entire PACE and IACE datasets and positive and negative effects for each position were summed separately. The positive and negative effect sums were then scaled by dividing by the maximum absolute value across all positions, yielding way to estimate which positions have the most potential to increase or decrease binding through mutation.

#### Variant selection for experimental testing.

Predictions from PEx_NN, PACE_NN, and PAnti_NN were combined to identify RBD variants predicted to preserve ACE2 binding while exhibiting altered antibody-escape potential. High-scoring variants were prioritized for synthesis and testing in the I3 experimental round. These selected variants provided a direct test of the model’s predictive power and informed subsequent model retraining and refinement.

#### Variant selection criteria and design of experimental library.

Potential variants were selected for further experiments using a variety of criteria. After eliminating variants with either lower predicted ACE2 binding or lower predicted expression than their parent strain, 25 variants of Omicron BA.1 and 25 variants of Omicron BA.5 with the highest predicted mean Log_10_(binding escape score) were selected. The top five Omicron BA.1 variants also had their mutations applied to Omicron BA.5 and vice versa. Ten single mutations were selected from each of the two Omicron strains by summing the mean Log_10_(binding escape score) for every variant in which the mutation appeared where expression and ACE2 binding was greater or equal to its parent strain. This was done to account for the quantity and severity of variants in which they appeared. Additionally, every pairwise combination of the ten single mutations from BA.1 was included and every pairwise combination of the ten single mutations from BA.5 was included. Each of the twenty total single mutations from both strains was also applied individually to the WT strain to create twenty new variants. Eleven SARS-CoV-2 strains (WT, Alpha, Beta, Delta, Epsilon, Gamma, Lambda, Kappa, Mu, Omicron BA.1 and Omicron BA.5) were included for comparison. Finally, any Omicron BA.1 or Omicron BA.5 single mutations relative to WT and present in the Greaney et al. [12] dataset were included for replication. Sixteen duplicate sequences were removed, leaving 213 total variants selected for further experimentation. These selected 213 RBD variants were produced in HEK cells, and their binding affinities with ACE2 and the 5 representative HCAbs from our in-house library of cross-variant anti-SARS-CoV-2 neutralizing HCAbs were measured [13] (S1 Data). A summary of variant categories and counts is provided in S2 Table.

### Experimental validation

#### Protein production of SARS-CoV-2 variant RBDs.

DNA fragments encoding SARS-CoV2 RBD variants were synthesized and cloned by Twist Bioscience Inc. Key features of these constructs include a 5’ KOZAK sequence before the start site, signal peptide, and sequence encoding a 10xHistidine tag and AVI tag at C-terminal. These plasmids were transformed into and amplified in 10B *Escherichia coli* cells (NEB, C3019H), extracted via Plasmid Plus 96 miniprep kits (Qiagen) and sequence verified by Genewiz (Azenta Life Sciences). These plasmids were then used to co-transfect with a BirA plasmid (Addgene, 64395) into Expi293F cells (Thermofisher Scientific, A14527) for *in situ* biotinylation. For *in situ* biotinylation, 10% by weight of the BirA plasmid was added for co-tranfection with SARS-CoV2 RBD plasmid at a ratio of 1ug of total plasmid for 1 mL of Expi293F cells. For example, a 5 mL Expi293F transfection used 0.5 µg of BirA plasmid and 4.5ug of SARS-CoV2 RBD plasmid. 5 ml culture for each variant were transfected in 24-deep-well plates and incubated at 37 ˚C 8% CO_2_ at 600 rpm with orbital diameter at 3mm. Transfection enhancers were added 18–22 hours post-transfection following manufacturer’s recommendations. 6 days post transfection, transfected cultures were harvested by centrifugation at 3000xg 4˚C for 10 minutes, then supernatants were filtered through a 0.22 µm membrane, concentrated via 3 kDa Amicon filters (Merck, UFC5003), buffer exchanged with 1xPBS at pH 7.2, and stored at 4˚C until use in ELISAs.

#### Heavy chain-only antibody enzyme-linked immunosorbent assays (HCAb ELISA).

SARS-CoV-2 RBDs were added at an approximate concentration of unpurified sups at 20x concentration and diluted 1:50 in coating buffer (100mM NaHCO_3_, 150mM NaCl, at pH 8.3) to Pierce Streptavidin Coated High Capacity 384 well clear Plates (Thermofisher Scientific, 15504) and incubated with a lid at room temperature for 1 hour at 400RPM on an orbital shaker. The plates were washed six times with a wash buffer (1xPBS/ 0.05% Tween-20 in Distilled H_2_O) by a EL406 plate washer (Agilent, Biotek), a step repeated between all subsequent steps. Plates were blocked with 50 µL/well Pierce Protein-Free Blocking buffer (Thermofisher Scientific, 37572) for 2 hours on an orbital shaker at 400 RPM at room temperature. Post-washing, experimental wells were incubated with dilutions of primary antibodies, HCAbs from 10µg/mL diluted 1:3 in blocking buffer for 2 hours on an orbital shaker 400 RPM at room temperature. After washing, a secondary antibody Horse radish peroxidase (HRP) conjugated Goat Anti-Human IgG (Thermofisher Scientific, 31412) was diluted 1:15000 in blocking buffer and then incubated in each well for 1 hour on an orbital shaker at 400 RPM at room temperature. After a final wash, plates were developed with 25µL/well of Ultra TMB (Thermofisher Scientific, 34028) for 5–7 minutes and the chromatic reaction was stopped with 25 µL 2M H_2_SO_4_. Plate wells were measured for absorbance at 450 nm on a Tecan Spark Cyto and analyzed to determine dissociation constants (K_D_) based on their titration curves.

#### Gyros HCAb-ACE2 competition assay.

Competition assays between top candidate HCAbs and ACE2-rbFc (rabbit Fc domain) fusion protein [17] were carried out on the Gyrolab xPlore system in Bioaffy 1000 HC CDs (Gyros Protein Technologies, P0020667). Samples were diluted in Rexxip A buffer (Gyros Protein Technologies, P0004820) and run using the General PK program. Biotinylated SARS-CoV-2 variant RBDs were used as the capture reagent at 10 µg/ml. A seven-point dilution series of HCAb (starting at 20 µg/ml; diluted 1:5 down) was pre-mixed with ACE2-rbFc (0.2 µg/ml in all samples & blank), and AlexaFluor 647 goat anti-rabbit IgG (H + L) (Invitrogen, A-21244) diluted to 2 ug/mL in Rexxip F buffer (Gyros Protein Technologies, P0004825) was used for detection.

#### *Calculation of experimental dissociation constants (K*_*D*_).

All binding dissociation constants were fitted using maximum likelihood estimation to a version of the Michaelis-Menten (MM) equation with background and Log-scaled concentrations:


$$\begin{array}{c} B=\frac{B_{max}}{\text{1}+{\text{1}\text{0}}^{K_{d}-x}}+bkg \end{array}$$

where B is the binding, $B_{max}$ is the maximum binding level, $K_{d}$ is the dissociation constant (the concentration at which $B=\frac{\text{1}}{\text{2}}B_{max}$), x is the concentration, bkg is the measurement background, and x and $K_{d}$ are in units of Log_10_(µg/mL). The Log-likelihood equation to be minimized was:


$$\begin{array}{c} \sum_{i}\text{ln}\left(t_{\frac{B_{i}-{\hat{B}}_{i}(\theta )}{\sigma },\text{4}}\right)-\text{ln}(\sigma ) \end{array}$$

where $t_{\alpha ,n}$ is the t distribution with n degrees of freedom evaluated at α, $B_{i}$ are the observed binding values, ${\hat{B}}_{i}(\theta )$ are the fitted binding values for parameter set θ, and σ is a fitted error term. A four degree of freedom t distribution was used to increase robustness and account for the long tails of the experimental data. Optimization was performed using a constrained Nelder-Mead algorithm with $\text{0}\leq B_{max}\leq \text{2}\text{max}(B)$, $\text{min}(x)-\text{1}\leq K_{d}\leq \text{max}(x)+\text{2}$, and 0≤bkg≤1. A constant background model, B=bkg, was also fitted, and the Akaike Information Criterion (AIC) was computed for each model. The model with the lowest AIC was chosen as the best, and data points where the constant model was chosen were discarded from further modeling.

## Supporting information

S1 Data 213 RBD variants and predicted metrics. (XLSX)

S1 Table Number of added and total unique datapoints in each successive model using our own experiments. (DOCX)

S2 Table A summary of variant categories and counts. (DOCX)

S1 Fig Performance of machine learning models for predicting SARS-CoV-2 RBD functional outcomes. (DOCX)

S2 Fig Comparison between experimental and predicted binding affinities expressed as Log10(KD_variant/ KD_WT) for a test set consisting of one fifth of the unique variants from I3, and trained on I1, I2, and the remainder of I3. (DOCX)

S3 Fig Binding-escape score distributions for all tested HCAbs across prioritized SARS-CoV-2 RBD variants. Boxplots summarize the variant-level escape scores (log₁₀(KD,HCAb/ KD,ACE2)) for each HCAb in the panel.
(DOCX)

S4 Fig Comparison of predicted vs. observed Delta(Log₁₀(Kₐ) binding scores with the global epistasis Com_Epi model. (DOCX)
