# Supplementary material for: Combining machine learning and iterative experiments to keep pace with emerging viral variants of concern
Source: PLoS Comput Biol. 2026 Jun 17;22(6):e1014394. doi: 10.1371/journal.pcbi.1014394 (PMC13274873; doi:10.1371/journal.pcbi.1014394)
Supplement: S1 Table — (DOCX) [file pcbi.1014394.s002.docx]

S1 Table. Number of added and total unique datapoints in each successive model using our own experiments.

| Version | Added Antibodies | Total Unique Antibodies | Added Variants (Excluding WT) | | Total Unique Variants (Excluding WT) | | Added Datapoints | Total Unique Datapoints |
| --- | --- | --- | --- | --- | --- | --- | --- | --- |
| 1 | 58 | 58 | | 6 | | 6 | 236 | 236 |
| 2 | 29 | 58 | | 2 | | 7 | 57 | 280 |
| 3 | 5 | 58 | | 195 | | 195 | 927 | 1183 |
