# Supplementary material for: Combining machine learning and iterative experiments to keep pace with emerging viral variants of concern
Source: PLoS Comput Biol. 2026 Jun 17;22(6):e1014394. doi: 10.1371/journal.pcbi.1014394 (PMC13274873; doi:10.1371/journal.pcbi.1014394)
Supplement: S2 Table — (DOCX) [file pcbi.1014394.s003.docx]

S2 Table: A summary of variant categories and counts

| **Variant category** | **Description** | **Count** |
| --- | --- | --- |
| Omicron BA.1 variants | Top 25 predicted escape variants | 25 |
| Omicron BA.5 variants | Top 25 predicted escape variants | 25 |
| Cross-lineage variants | Top 5 BA.1 mutations introduced into BA.5 and vice versa | 10 |
| BA.1 single mutations | Ten most frequent adaptive mutations (evaluated individually) | 10 |
| BA.5 single mutations | Ten most frequent adaptive mutations (evaluated individually) | 10 |
| Pairwise combinations (BA.1) | All pairwise combinations of 10 BA.1 single mutations | 45 |
| Pairwise combinations (BA.5) | All pairwise combinations of 10 BA.5 single mutations | 45 |
| WT + single mutations | Each of 20 single mutations introduced into WT background | 20 |
| Reference strains | WT + 10 WHO lineages (Alpha–BA.5) | 11 |
| Replication variants (Greaney *et al.* overlap) | WT-relative single mutations from BA.1/BA.5 | 12 |
| **Total (after removing 16 duplicates)** |  | **213** |
