# Supplementary material for: Combining machine learning and iterative experiments to keep pace with emerging viral variants of concern
Source: PLoS Comput Biol. 2026 Jun 17;22(6):e1014394. doi: 10.1371/journal.pcbi.1014394 (PMC13274873; doi:10.1371/journal.pcbi.1014394)
Supplement: S1 Fig — (DOCX) [file pcbi.1014394.s004.docx]

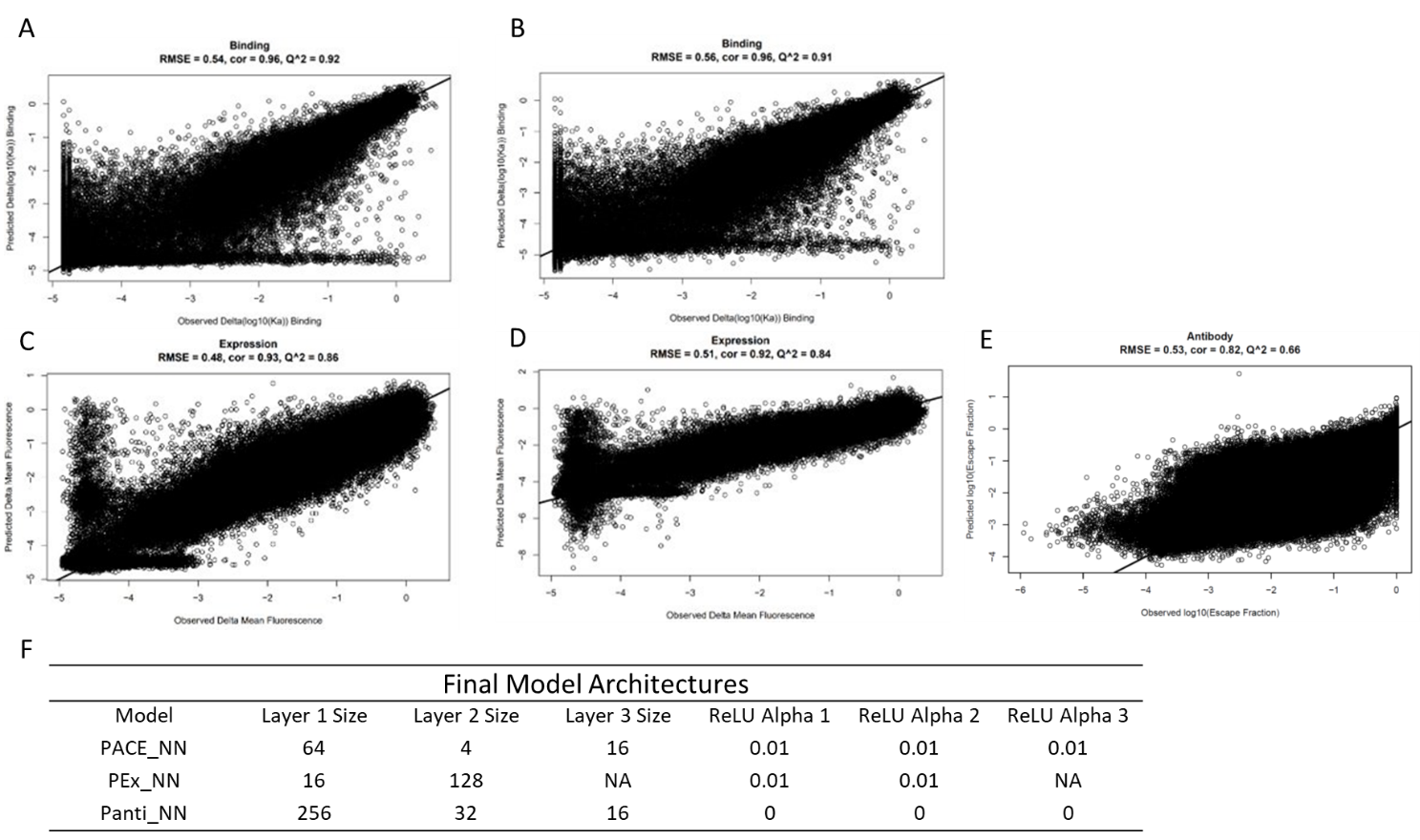


S1 Fig. **Performance of machine learning models for predicting SARS-CoV-2 RBD functional outcomes.** (A–B) PACE_NN: Predicted vs. observed log_10_(K_D_variant_/K_D_WT_) binding scores for (A) the final tuned model with performance metrics: Corr = 0.96, Q^2^ = 0.92, RMSE = 0.54 and (B) when the tuning process occurs separately inside each CV fold. Validation method (B) estimates a slightly lower accuracy at: Corr = 0.96, Q^2^ = 0.91, RMSE = 0.56. (C–D) PEx_NN: Predicted vs. observed Δ mean fluorescence values (a proxy for expression) for (C) the final tuned model with performance metrics: Corr = 0.93, Q^2^ = 0.86, RMSE = 0.48 and (D) when the tuning process occurs separately inside each CV fold. Validation method (D) estimates a slightly lower accuracy at: Corr = 0.92, Q^2^ = 0.84, RMSE = 0.51. (E) PAnti_NN: Predicted vs. observed Log_10_(Escape Fraction) for the final tuned model demonstrates moderate-to-strong predictive accuracy (Corr = 0.82, Q² = 0.66, RMSE = 0.53) and (F) parameters for final tuned model architectures.
