# Supplementary material for: Combining machine learning and iterative experiments to keep pace with emerging viral variants of concern
Source: PLoS Comput Biol. 2026 Jun 17;22(6):e1014394. doi: 10.1371/journal.pcbi.1014394 (PMC13274873; doi:10.1371/journal.pcbi.1014394)
Supplement: S2 Fig — (DOCX) [file pcbi.1014394.s005.docx]

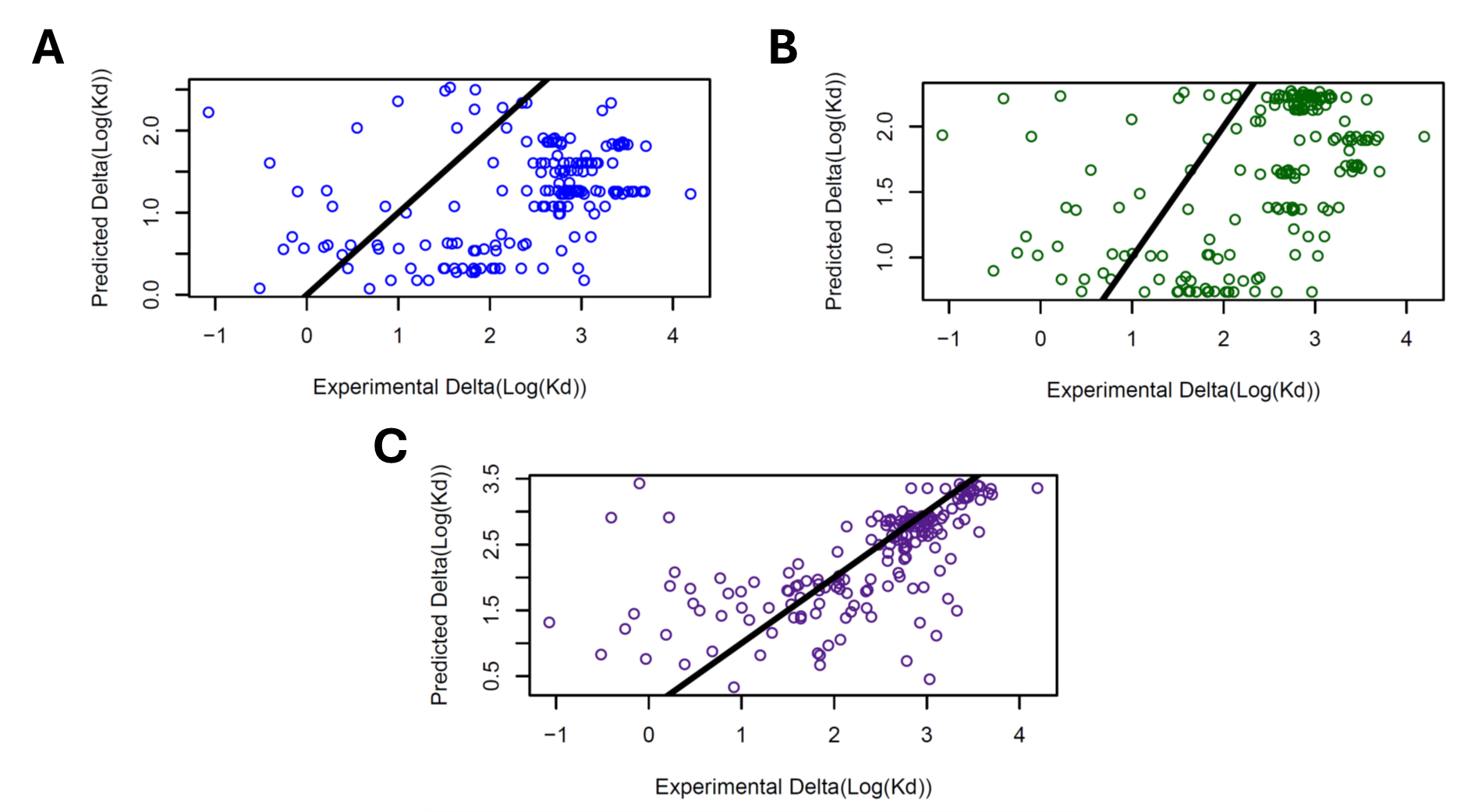
 S2 Fig. **Comparison between experimental and predicted binding affinities expressed as Log10(KD_variant / KD_WT) for a test set consisting of one fifth of the unique variants from I3, and trained on I1, I2, and the remainder of I3.** (A) I1_RF: trained on binding data for 59 newly characterized HCAbs against the Beta, Delta, Gamma, Lambda, Mu, Omicron BA.1, and WT variants (RMSE = 1.51; Corr = 0.37; Q² = -1.28). (B) I2_RF: extended to 15 antibodies tested against Omicron BA.1, Omicron BA.5, and WT (RMSE = 1.16; Corr = 0.46; Q² = -0.35). (C) I3_RF: final model including data on five representative HCAbs evaluated across 174 remaining RBD variants (RMSE = 0.78; Corr = 0.64; Q² = 0.39). I1_RF and I2_RF consistently make predictions that are too low, probably because the I3 dataset has a much higher median Log10(KD_variant / KD_WT) at 2.61, compared to 0.23 and 0.31 for I1 and I2, respectively. However, predicted and experimental values are still positively correlated in all cases.
