# Supplementary material for: Combining machine learning and iterative experiments to keep pace with emerging viral variants of concern
Source: PLoS Comput Biol. 2026 Jun 17;22(6):e1014394. doi: 10.1371/journal.pcbi.1014394 (PMC13274873; doi:10.1371/journal.pcbi.1014394)
Supplement: S3 Fig — Boxplots summarize the variant-level escape scores (log₁₀(KD,HCAb/ KD,ACE2)) for each HCAb in the panel. (DOCX) [file pcbi.1014394.s006.docx]

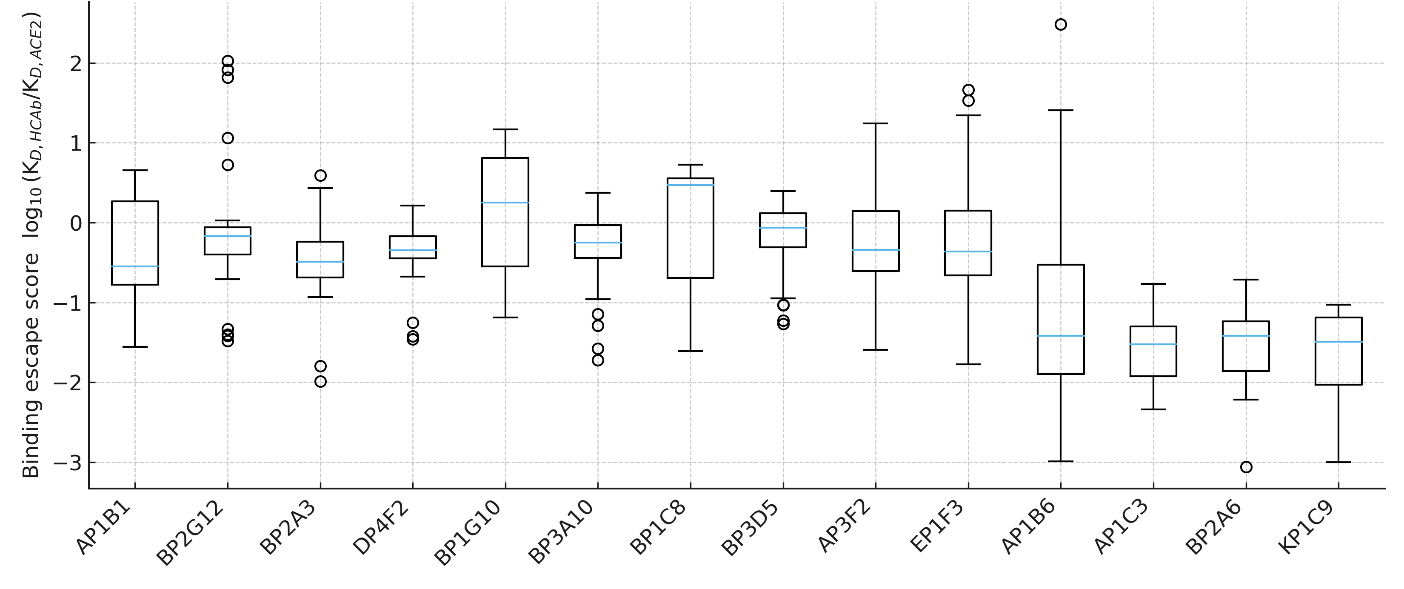


S3 Fig. **Binding-escape score distributions for all tested HCAbs across prioritized SARS-CoV-2 RBD variants.** Boxplots summarize the variant-level escape scores (log₁₀(KD,HCAb / KD,ACE2)) for each HCAb in the panel.
