# Supplementary material for: Combining machine learning and iterative experiments to keep pace with emerging viral variants of concern
Source: PLoS Comput Biol. 2026 Jun 17;22(6):e1014394. doi: 10.1371/journal.pcbi.1014394 (PMC13274873; doi:10.1371/journal.pcbi.1014394)
Supplement: S4 Fig — (DOCX) [file pcbi.1014394.s007.docx]

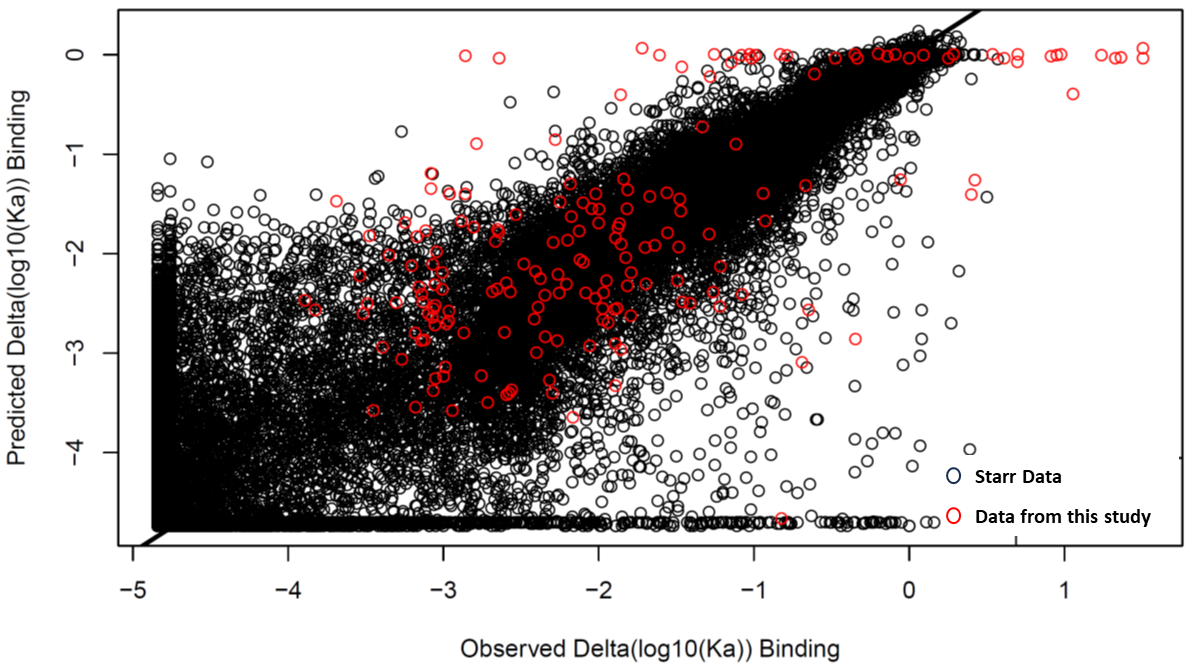


S4 Fig. **Comparison of predicted vs. observed Delta(Log₁₀(Kₐ) binding scores with the global epistasis Com_Epi model.** Scatterplot showing predicted versus observed binding affinities log_10_(K_D_variant_/K_D_WT_) for SARS-CoV-2 RBD variants. Data points are colored by source: black circles represent the PACE dataset; red circles indicate measurements from the I3 dataset. Performance metrics for the combined datasets: RMSE = 0.55, Corr = 0.96, Q^2^ = 0.92. Performance metrics for the I3 dataset alone: RMSE = 0.98, Corr = 0.65, Q^2^ = 0.36.
